# G-protein Gα_13_ functions as a cytoskeletal and mitochondrial regulator to restrain osteoclast function

**DOI:** 10.1038/s41598-019-40974-z

**Published:** 2019-03-12

**Authors:** Shinichi Nakano, Kazuki Inoue, Cheng Xu, Zhonghao Deng, Viktoriya Syrovatkina, Gregory Vitone, Liang Zhao, Xin-Yun Huang, Baohong Zhao

**Affiliations:** 1 Arthritis and Tissue Degeneration Program and The David Z, Rosensweig Genomics Research Center, Hospital for Special Surgery, New York, New York, USA; 2 Department of Medicine, Weill Cornell Medical College, New York, New York, USA; 3 Department of Orthopedic Surgery, Nanfang Hospital, Southern Medical University, Guangzhou, Guangdong, China; 4 Department of Physiology and Biophysics, Weill Cornell Medical College, New York, New York, USA; 5 Graduate Program in Cell & Developmental Biology, Weill Cornell Graduate School of Medical Sciences, New York, New York, USA

## Abstract

Excessive osteoclastic bone erosion disrupts normal bone remodeling and leads to bone loss in many skeletal diseases, including inflammatory arthritis, such as rheumatoid arthritis (RA) and psoriatic arthritis, periodontitis and peri-prosthetic loosening. Functional control of osteoclasts is critical for the maintenance of bone homeostasis. However, the mechanisms that restrain osteoclast resorptive function are not fully understood. In this study, we identify a previously unrecognized role for G-protein Gα_13_ in inhibition of osteoclast adhesion, fusion and bone resorptive function. Gα_13_ is highly expressed in mature multinucleated osteoclasts, but not during early differentiation. Deficiency of Gα_13_ in myeloid osteoclast lineage (*Gα*_*13*_^*ΔM/ΔM*^ mice) leads to super spread morphology of multinucleated giant osteoclasts with elevated bone resorptive capacity, corroborated with an osteoporotic bone phenotype in the *Gα*_*13*_^*ΔM/ΔM*^ mice. Mechanistically, Gα_13_ functions as a brake that restrains the c-Src, Pyk2, RhoA-Rock2 mediated signaling pathways and related gene expressions to control the ability of osteoclasts in fusion, adhesion, actin cytoskeletal remodeling and resorption. Genome wide analysis reveals cytoskeleton related genes that are suppressed by Gα_13_, identifying Gα_13_ as a critical cytoskeletal regulator in osteoclasts. We also identify a genome wide regulation of genes responsible for mitochondrial biogenesis and function by Gα_13_ in osteoclasts. Furthermore, the significant correlation between Gα_13_ expression levels, TNF activity and RA disease activity in RA patients suggests that the Gα_13_ mediated mechanisms represent attractive therapeutic targets for diseases associated with excessive bone resorption.

## Introduction

Bone destruction is a major cause of disability associated with many skeletal diseases, such as rheumatoid arthritis (RA), psoriatic arthritis, periodontitis and peri-prosthetic loosening^1–5^. Osteoclasts are the exclusive cell type that is responsible for bone resorption. Excessive generation of osteoclasts or increased osteoclast activity leads to pathologic bone erosion. Osteoclasts differentiate from the monocyte/macrophage lineage. Osteoclastogenesis is induced by the master osteoclastogenic cytokine receptor activator of nuclear factor-κB ligand (RANKL), which acts in concert with macrophage colony-stimulating factor (M-CSF) and immunoreceptor tyrosine-based activation motif (ITAM)-mediated co-stimulatory signals. Binding of RANKL to its receptor RANK activates a broad range of signaling cascades, including canonical and non-canonical NF-κB pathways; mitogen-activated kinase (MAPK) pathways leading to the activation of AP-1 and CREB transcription factors; and calcium signaling, to induce the expression of key transcription factors Blimp1 and NFATc1 to initiate early stage of osteoclast differentiation^2,6–9^.

Along the differentiation process, myeloid precursors first undergo differentiation to mononuclear osteoclasts, which then fuse into mature giant multinucleated polykaryons at late stages driven by the expression of genes responsible for cell-cell fusion, such as DC-Stamp and ATP6v0d2^10,11^. The giant multinucleated osteoclasts express high levels of osteoclast marker genes, such as Tartrate Resistant Acidic Phosphatase (*Acp5*), Cathepsin K (*CtsK*) and αvβ3 integrin (*Itgb3*). The expression of these genes is essential for mature osteoclasts to exert their major function, bone-resorption, through acid decalcification and proteolytic degradation of the bone matrix^2,9^. As a premise, the process of bone resorption requires osteoclasts to be polarized and to adhere tightly to the bone surface to form a unique structure, called the sealing zone, via actin ring formation surrounding a ruffled border. The unique osteoclastic actin ring structure makes the sealing zone an isolated resorptive microenvironment that concentrates the secreted protons and degrading enzymes for efficient resorption to occur^12–14^. Upon stimulation, such as attachment to integrins and/or cytokine activation by M-CSF or RANKL, osteoclasts undergo rapid cytoskeletal reorganization, appear polarized and form actin rings (the actin rings display as actin rings/belts in *in vitro* cultures)^14,15^. c-Src initiated signaling cascades including downstream activation of Syk, Pyk2 and small Rho GTPases are well studied and known to play important roles in actin reorganization^14,15^. Cytoskeletal reorganization in osteoclasts, mainly reflected by dynamic actin ring formation, is a prerequisite for osteoclast polarization, movement and bone resorptive function. Recent evidence shows that functional control of osteoclasts is important for the maintenance of bone homeostasis^16–19^. Nonetheless, the mechanisms regulating osteoclast cytoskeleton organization and function, especially the negative feedback mechanisms, remain underexplored.

G-protein mediated signaling pathways contribute to diverse cellular activities, such as cell growth, differentiation and survival. Guanine nucleotide-binding protein subunit alpha 13 (Gα_13_; encoded by *Gna13*) is a member of the G12 subfamily of the heterotrimeric G proteins. These G-proteins include Gα, Gβ, and Gγ subunits and couple specific receptors to transduce signals to downstream effectors^20,21^. The G12 subfamily has two members, Gα_12_ and Gα_13_. Gα_13_ is more critical than Gα_12_ during development, demonstrated by the genetic evidence that mice with global knockout of Gα_13_, but not Gα_12_, exhibit embryonic lethality^22,23^. Gα_13_ plays key roles in biological and disease settings, such as embryogenesis, angiogenesis, cell cytoskeleton organization, cellular transformation, and metastatic tumor progression^21^. In addition, Gα_13_ can interact with other proteins to regulate different signaling pathways, such as MAPK pathway and the Rho GTPases^20^. Wu *et al*. recently reported that Gα_13_ negatively regulates osteoclast differentiation through inhibition of the Akt-GSK3β-NFATc1 signaling^24^. Here, we identified a previously unrecognized expression pattern for Gα_13_ and unique molecular mechanisms by which Gα_13_ regulates osteoclast function. In contrast to a low expression level of Gα_13_ in osteoclast precursors and early stages of differentiation, we found that Gα_13_ is highly expressed in multinucleated osteoclasts, indicating a potential role for Gα_13_ in mature osteoclasts. Given the critical roles for G12 proteins in regulating cytoskeleton in fibroblasts and endothelial cells^25–28^, it would be of interest and importance to explore whether Gα_13_ regulates osteoclast cytoskeletal reorganization and function.

In the present study, we found that Gα_13_ is a RANKL-inducible G-protein that is highly expressed in multinucleated osteoclasts and plays an important feedback inhibitory role in controlling osteoclast actin ring formation and resorptive function. We found that Gα_13_ deficiency in myeloid lineage does not affect early stages of osteoclast differentiation, but enhances gene expression responsible for osteoclast fusion, resorption and cytoskeletal reorganization. The inducible Gα_13_ expression pattern in multinucleated osteoclasts may explain its stage-dependent function. Compared to the wild type (WT) cells, the Gα_13_ conditional knockout (KO) osteoclasts show super spread giant multinucleated morphology both *in vitro* and *in vivo* with rapid actin ring reorganization and increased bone resorption. Osteoclast cytoskeletal organization and resorption is a highly energy consuming process. The enhanced osteoclast function was further corroborated by highly enriched expression of genes involved in cytoskeleton, mitochondrial biogenesis and function in Gα_13_ conditional KO osteoclasts by genome wide analysis of gene expression and Gene Oncology (GO) analysis. Collectively, our findings uncovered a novel function of Gα_13_ that acts as a cytoskeletal and mitochondrial regulator to play a feedback inhibitory role in osteoclast fusion, cytoskeletal reorganization and function. Furthermore, we found that Gα_13_ expression is inversely correlated with TNF and RA disease activity, suggesting that appropriate modulation of Gα_13_ would provide an alternative strategy to control osteoclast function thereby preventing bone loss.

## Results

### Gα_13_ is a RANKL inducible gene that controls osteoclast size and resorptive function

**Figure 1:**
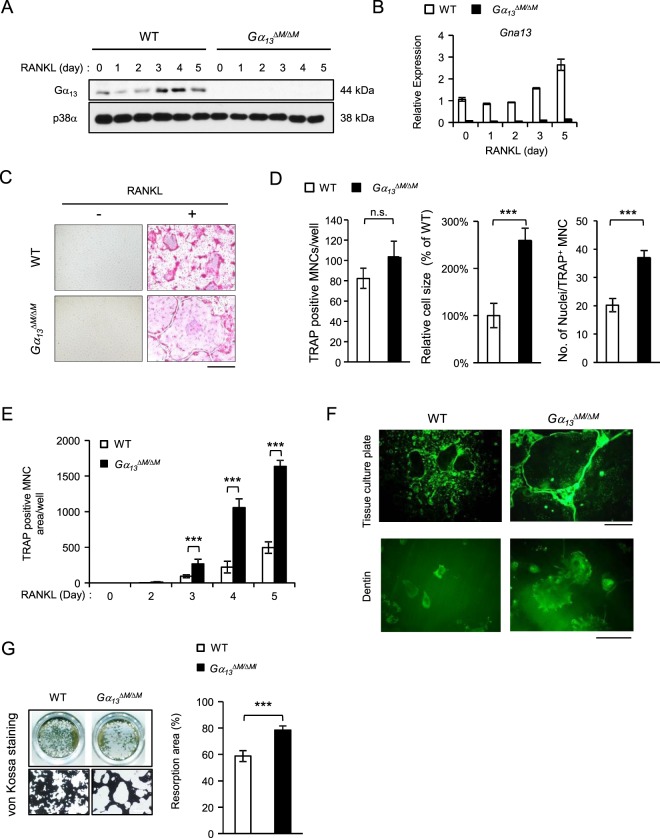
Gα_13_ is a RANKL inducible inhibitor that restrains osteoclast size and resorptive activity. (**A**) Immunoblot analysis and (**B**) quantitative real-time PCR analysis of mRNA *(Gna*13*)* expression of Gα_13_ induced by RANKL. (**C**) Osteoclast differentiation using BMMs derived from WT and *Gα*_*13*_^*ΔM/ΔM*^ mice stimulated with RANKL for five days. TRAP staining was performed and TRAP-positive multinucleated cells (MNCs) appear red in the photographs. Scale bar, 500 µm. (**D**) The number (left panel) and the relative average size (middle panel) of TRAP-positive MNCs (> = 3 nuclei per cell) per well and the average numbers of nuclei per TRAP-positive MNC (right panel) were quantified by Osteomeasure software. (**E**) Quantification of the area of TRAP positive MNCs derived from WT and *Gα*_*13*_^*ΔM/ΔM*^ at the indicated days in the presence of RANKL (40 ng/ml). (**F**) Upper panel: Actin ring formation in the cell culture plates of BMMs derived from WT and *Gα*_*13*_^*ΔM/ΔM*^ mice stimulated with RANKL for five days. Podosome belts are peripherally located. Scale bar, 500 μm. Lower panel: Actin ring formation on dentin slices of BMMs derived from WT and *Gα*_*13*_^*ΔM/ΔM*^ mice stimulated with RANKL for six days. Actin rings are peripherally or internally located. Scale bar, 100 μm. (**G**) Mineral resorption activity of MNCs derived from WT and *Gα*_*13*_^*ΔM/ΔM*^ mice. The BMMs were cultured on the calcium coated plates with RANKL for seven days. Von kossa staining was performed to detect resorptive pit areas. The representative images of whole well (upper) and 10× magnified field (lower) were shown in the left panel. Scale bars = 500 μm. Quantification of resorptive area relative to whole well area was shown in the right panel. All data are shown as mean ± S.D. ***p < 0.001, n.s., not statistically significant.

**Figure 2:**
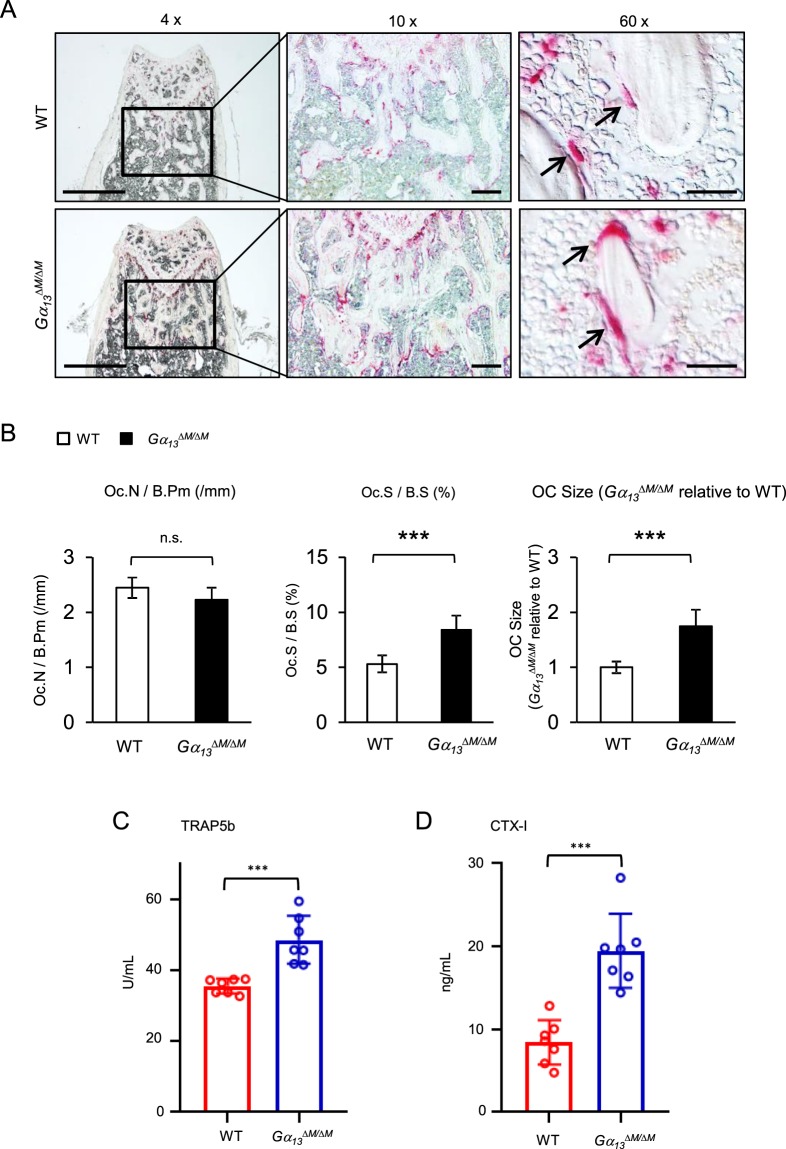
Gα_13_ deficiency increases osteoclast size but not numbers *in vivo*. TRAP staining (**A**) and histomorphometric analysis (**B**) of histological sections obtained from the metaphysis region of distal femurs of two-month-old male *Gα*_*13*_^*ΔM/ΔM*^ mice and their littermate WT control mice. Scale bars = 1 mm (4 × objective), 200 μm (10 × objective) or 20 μm (60 × objective) respectively. Oc.S/BS, osteoclast surface per bone surface; Oc. N/B.Pm, number of osteoclasts per bone perimeter. OC size, osteoclast surface divided by osteoclast number. Serum TRAP (**C**) and CTX-I (**D**) levels measured in the serums obtained from 8–12 week old age and gender both matched *Gα*_*13*_^*ΔM/ΔM*^ mice and their littermate WT control mice. n = 7. All data are shown as mean ± S.D. ***p < 0.001, n.s., not statistically significant.

**Figure 3:**
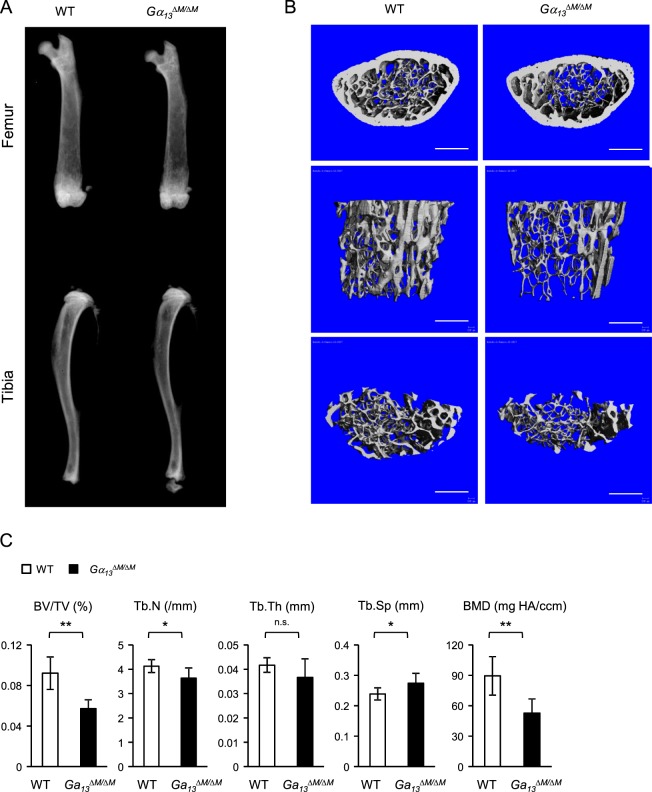
*Gα*_*13*_^*ΔM/ΔM*^ mice exhibit osteoporotic phenotype. (**A**) Radiographs of femurs and tibia, (**B**) μCT images and (**C**) bone morphometric analysis of the trabecular bone of the distal femurs isolated from two-month-old WT and *Gα*_*13*_^*ΔM/ΔM*^ male mice. Scale bar = 500 μm. n = 6 per group. All data are shown as mean ± S.D. *p < 0.05, **p < 0.01, n.s., not statistically significant. BV/TV, bone volume per tissue volume; Tb.N, trabecular number; Tb.Th, trabecular bone thickness; Tb.Sp, trabecular bone spacing, BMD, bone mineral density.

Global deletion of Gα_13_ expression in mice leads to embryonic lethality^22,23^. Thus, to determine the role of Gα_13_ in osteoclastogenesis, we deleted *Gna13* (encoding Gα_13_) in myeloid lineage osteoclast precursors by crossing *Gα*_*13*_^*flox/flox*^ mice with *LysMcre* mice that express Cre under the control of the myeloid-specific lysozyme M promoter. We used *Gα*_*13*_^*flox/flox*^*LysMcre*(+) mice (hereafter referred to as *Gα*_*13*_^*ΔM/ΔM*^) and littermate controls with a *Gα*_*13*_^+/+^ *LysMcre*(+) genotype (hereafter referred to as wild type (WT)) in the experiments. The bone marrow derived macrophages (BMMs) were used as osteoclast precursors *in vitro*. We first examined the mRNA and protein expression levels of Gα_13_ during osteoclast differentiation induced by RANKL, a master osteoclastogenic cytokine. We observed that low Gα_13_ expression levels were maintained throughout the early stage of differentiation and were not highly induced until three days after RANKL stimulation, when the cells generally started fusion and became mature multinucleated giant osteoclasts in cultures (Fig. 1A,B). This inducible Gα_13_ expression pattern indicates its potential dominant role presumably in the late stage of osteoclast differentiation. Indeed, we found that *Gα*_*13*_^*ΔM/ΔM*^ derived BMMs formed nearly similar number of TRAP-positive multinucleated osteoclasts, but strikingly increased the average osteoclast area to approximately three times of that of the WT cells (Fig. 1C,D). The average numbers of nuclei per TRAP-positive MNC in *Gα*_*13*_^*ΔM/ΔM*^ osteoclasts were significantly greater than those in WT osteoclasts (Fig. 1D), indicating a suppressive role for Gα_13_ in cell fusion. The *Gα*_*13*_^*ΔM/ΔM*^ osteoclast area began to increase after three days of RANKL stimulation and increased more drastically after four or five days of RANKL stimulation (Fig. 1E). The super spread *Gα*_*13*_^*ΔM/ΔM*^ multinucleated osteoclasts formed F-actin rings (podosome belts) on both tissue culture plates and dentin slices (Fig. 1F) and were functional in mineral resorption (Fig. 1G). Consistent with the osteoclast area, the resorptive area generated by the *Gα*_*13*_^*ΔM/ΔM*^ cells was much larger than that by the WT osteoclasts (Fig. 1G). Notably, the super spread giant osteoclasts were also observed in the *Gα*_*13*_^*ΔM/ΔM*^ mice (Fig. 2A). Similarly as in the *in vitro* cultures, Gα_13_ deficiency did not result in different osteoclast numbers *in vivo*. Significantly, the average osteoclast size and the relative osteoclast surface to bone surface were both enhanced in the *Gα*_*13*_^*ΔM/ΔM*^ mice (Fig. 2B). We furthermore examined the serum levels of TRAP and C-terminal telopeptide of type 1 collagen (CTX-I), which are important indicators reflecting osteoclast activity *in vivo*. As shown in Fig. 2C,D, both serum TRAP and CTX-I levels were significantly elevated in the *Gα*_*13*_^*ΔM/ΔM*^ mice. We next performed microcomputed tomographic (μCT) analyses in order to examine whether the super spread *Gα*_*13*_^*ΔM/ΔM*^ osteoclasts have effects on the bone phenotype. As shown in Fig. 3, *Gα*_*13*_^*ΔM/ΔM*^ mice exhibit the osteoporotic phenotype indicated by decreased trabecular bone volume, number, bone mineral density and increased trabecular bone spacing. Taken together with the enhanced osteoclast surface and size *in vivo* in *Gα*_*13*_^*ΔM/ΔM*^ mice, these data demonstrate that the resorptive function of the super spread *Gα*_*13*_^*ΔM/ΔM*^ osteoclasts is significantly enhanced, leading to an osteoporotic bone phenotype. Collectively, our results identify a previously unrecognized role for Gα_13_ that controls the size of osteoclasts during the late stage of osteoclast differentiation. Deficiency of Gα_13_ leads to super spread morphology of multinucleated giant osteoclasts with elevated bone resorptive capacity.

### Gα_13_ deficiency affects the expression of genes responsible for osteoclast terminal differentiation and function

**Figure 4:**
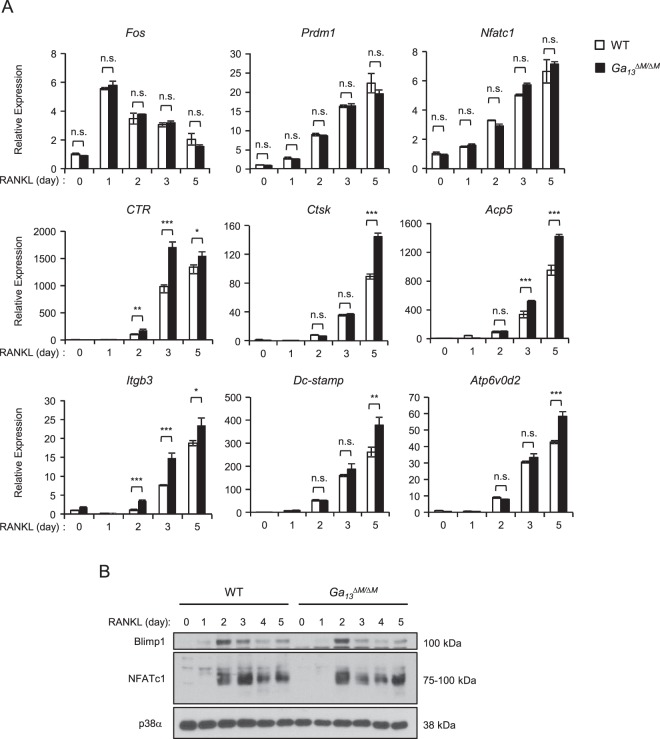
Gα_13_ selectively suppresses the expression of genes responsible for osteoclast function and fusion, but not for early differentiation. (**A**) Quantitative real-time PCR analysis of mRNA expression of *Fos (encoding c-Fos)*, *Prdm1* (encoding Blimp1), *Nfatc1* (encoding NFATc1), *CTR* (encoding calcitonin receptor), *Ctsk* (encoding cathepsin K), *Acp5* (encoding TRAP), *Itgb3* (encoding integrin β3), *Dc-stamp* (encoding DC-stamp) and *Atp6v0d2* (encoding ATP6v0d2) in WT and *Gα*_*13*_^*ΔM/ΔM*^ osteoclast cultures induced by RANKL at the indicated times. (**B**) Immunoblot analysis of the expression of Blimp1 and NFATc1 in WT and *Gα*_*13*_^*ΔM/ΔM*^ osteoclast cultures induced by RANKL at the indicated times. p38α was used as a loading control. All data are shown as mean ± S.D. *p < 0.05, **p < 0.01, ***p < 0.001, n.s., not statistically significant.

We next investigated the molecular mechanisms by which Gα_13_ regulates osteoclast size and function. We first examined the expression of the genes that play key roles in osteoclast differentiation, fusion and resorptive function. Consistent with the phenotype that Gα_13_ does not significantly change osteoclast numbers, Gα_13_ deficiency did not alter the induction and expression levels of key osteoclastogenic genes induced by RANKL (Fig. 4A,B), such as *Fos* (encoding c-Fos), *Nfatc1* (encoding NFATc1) and *Prdm1* (encoding Blimp1), each of which is essential for early osteoclast differentiation. Furthermore, the early osteoclastogenic signaling pathways, such as NF-κB, MAPK and c-Fos, were activated similarly by RANKL between the WT and *Gα*_*13*_^*ΔM/ΔM*^ osteoclast precursors (data not shown). In addition, phosphorylation of AKT in response to M-CSF or RANKL was not affected by Gα_13_ (Supplementary Fig. 1). These results collectively support the phenotype that Gα_13_ does not affect early stage of osteoclast differentiation. On the other hand, the genes that control osteoclast function, such as *CTR* (encoding calcitonin receptor), *Ctsk* (encoding cathepsin K), *Acp5* (encoding TRAP) and *Itgb3* (encoding integrin β3), were significantly enhanced by RANKL in Gα13 deficient cell cultures (Fig. 4A). These findings provide a molecular basis for the enhanced bone resorptive function of Gα_13_ deficient osteoclasts. Cell fusion process regulates osteoclast size. Following this line, we found that the expression levels of the genes responsible for osteoclast fusion, *Dc-stamp* (encoding DC-STAMP) and *Atp6v0d2* (encoding ATP6v0d2), were higher in *Gα*_*13*_^*ΔM/ΔM*^ osteoclasts than WT cells (Fig. 4A). The negative regulation of these cell-cell fusion genes by Gα_13_ might be responsible, at least partially, for the super spread morphology of *Gα*_*13*_^*ΔM/ΔM*^ osteoclasts with large sizes.

### Gα_13_ regulates cytoskeletal reorganization in osteoclasts

**Figure 5:**
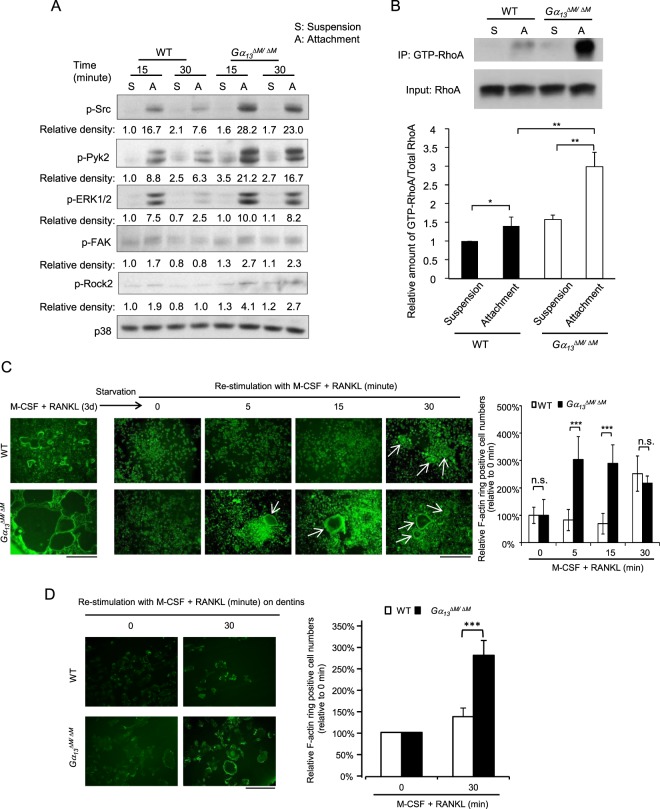
Gα_13_ plays an inhibitory role in actin cytoskeleton organization in osteoclasts. Osteoclast differentiation of WT and *Gα*_*13*_^*ΔM/ΔM*^ BMMs was induced by RANKL for three days, the cells were starved in the culture medium without FBS, M-CSF and RANKL for 5 hours, and then the cells were lifted and replated onto Fibronectin-coated dishes for 15 or 30 min (A: attachment) or left in suspension (S: suspension), followed by the immunoblot analysis (**A**) of the expression of phospho-Src (Tyr416), phospho-Pyk2 (Thy402), phospho-ERK1/2 (Thr202/Tyr204), phospho-FAK (pY397) and phospho-Rock2 (Ser1366). p38α was used as a loading control. The relative density of each band to its corresponding loading control p38 band was calculated by Image J software, and then was normalized to the WT suspension condition (the 1^st^ lane). (**B**) GTP-RhoA pulldown assay was performed using the 30 minute samples (a representative pulldown image, upper panel). The amount of GTP-bound RhoA was normalized to the total amount of RhoA (relative immunoblot band density) in cell lysates for the comparison of RhoA activity in different samples (lower panel). n = 3. Data are shown as mean ± S.D. *p < 0.05, **p < 0.01. (**C**,**D**) F-actin phalloidin staining (left panel) of non-starved cell cultures stimulated with M-CSF and RANKL for three days (C, on the tissue culture plates), or the cell cultures starved for five hours followed by re-stimulation with M-CSF and RANKL for the indicated times (C, on the tissue culture plates; D, on dentin slices). Scale bar in C, 500μm. Scale bar in D, 100 μm. Quantification of the relative numbers of F-actin ring positive cells to their corresponding time 0 condition was shown in the right panels. All data are shown as mean ± S.D. ***p < 0.001, n.s., not statistically significant.

In addition to gene expression, cytoskeletal reorganization, including cellular adhesion to bone surface and actin ring (podosome belt) formation to generate an isolated resorptive microenvironment (sealing zone), is essential for osteoclasts to effectively execute their bone resorbing function. Moreover, dynamic remodeling of actin cytoskeleton is critical for osteoclasts to cycle actively between resorption-migration-resorption phases on the bone surface. The integrin- or cytokine-mediated cytoskeletal organization is mainly activated via a canonical signaling pathway, which is initiated by c-Src activation. Therefore, we first examined the levels of phosphor-c-Src, an indicator of c-Src activity. To determine the role of Gα_13_ in the activation of c-Src in response to the integrin-mediated adhesion and signaling, we lifted and then re-plated WT or *Gα*_*13*_^*ΔM/ΔM*^ osteoclasts on culture dishes coated with fibronectin, which binds to cellular integrins and activates integrin-medicated signaling. We found that the phosphorylation of c-Src appeared much stronger in the *Gα*_*13*_^*ΔM/ΔM*^ cell cultures in response to adhesion than in the WT cultures, and was maintained at higher levels in the *Gα*_*13*_^*ΔM/ΔM*^ cell cultures (Fig. 5A). In addition, Pyk2 phosphorylation is critical for osteoclast adhesion, spreading and bone resorption. Phosphor-Erk and phosphor-FAK levels also play important roles in focal adhesion signaling and the regulation of osteoclast cytoskeletal reorganization^14,15,29^. Similarly as the regulation of c-Src activity, Gα_13_ deficiency significantly elevated the adhesion-induced phosphorylation levels of Pyk2, FAK and Erk (Fig. 5A). Next, we investigated whether Gα_13_ regulates the activity of Rho GTPases, the downstream effectors of these signaling pathways and central players in cytoskeleton reorganization. We found that Gα_13_ deficiency increased the GTP bound RhoA levels in response to osteoclastic adhesion (Fig. 5B), consistent with the upstream signaling enhancement. The GTP-Rac1 level was not much affected by the absence of Gα_13_ (data not shown). These results indicate that Gα_13_ is an inhibitor of RhoA activity during cytoskeletal remodeling in osteoclasts. Rho-associated kinase Rock2 is a key downstream effector of RhoA. In parallel to the changes of RhoA activity, the phosphorylation levels at S1366 of Rock2, an indicator of its activation, were enhanced by Gα_13_ deletion (Fig. 5A). These results further support an inhibitory role for Gα_13_ in controlling the RhoA-Rock2 pathway during cytoskeleton organization in osteoclasts.

Furthermore, we investigated whether Gα_13_ regulates dynamic remodeling of actin cytoskeleton. To address this question, we tested the cytokine-mediated actin ring re-organization of osteoclasts. The WT and *Gα*_13_^*ΔM/ΔM*^ osteoclast cultures were starved of M-CSF and RANKL. Five hours later, as expected with the knowledge of the importance of cytokine in cytoskeleton organization, the actin rings disappeared in both WT and *Gα*_*13*_^*ΔM/ΔM*^ osteoclast cultures on tissue culture plates or on dentin slices (Fig. 5C,D, time 0 after starvation). Upon re-stimulation with M-CSF and RANKL, Gα_13_ deficiency enabled the cells to reorganize the actin cytoskeleton to form actin rings within 5 minutes, which was much faster than the WT cells that needed 30 minutes (Fig. 5C). On dentin slices, reorganized actin rings formed significantly more in *Gα*_*13*_^*ΔM/ΔM*^ osteoclast cultures than in WT cultures after 30 minutes of restimulation with M-CSF and RANKL (Fig. 5D). These findings collectively indicate that Gα_13_ functions as a brake that restrains the signaling pathways mediated by Src, Pyk2, Erk, FAK and RhoA-Rock2, which are important for cytoskeleton remodeling, such as in the process of adhesion, actin reorganization and actin-ring formation. Gα_13_, a RANKL inducible inhibitor, thus plays a feedback inhibitory role in cytoskeletal reorganization in osteoclasts.

### Genome wide analysis reveals that Gα_13_ regulates key genes responsible for cytoskeleton, mitochondrial biogenesis and function

**Figure 6:**
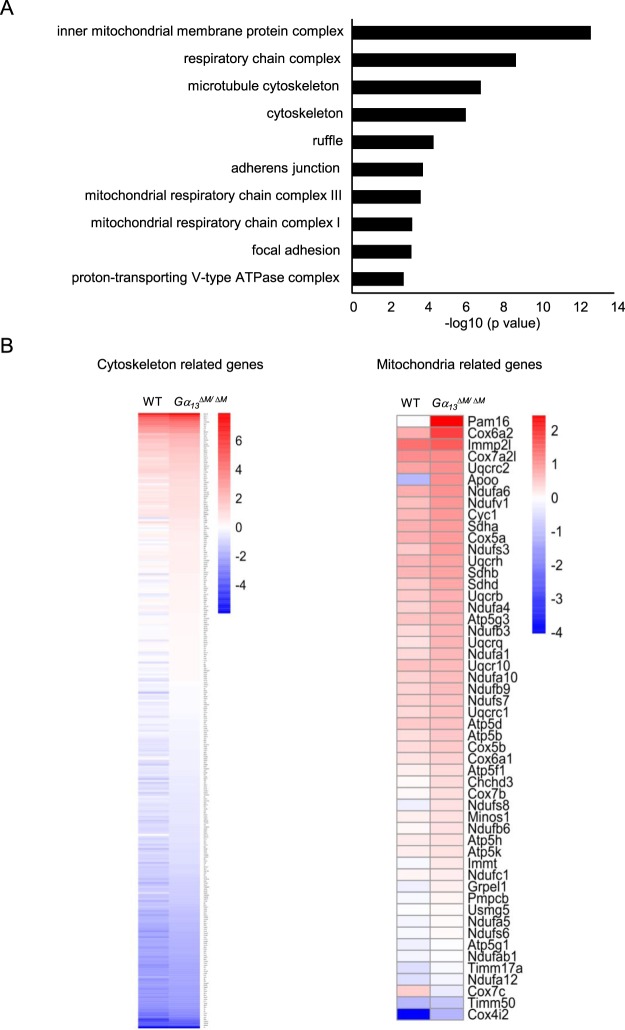
Genome wide analysis shows that Gα_13_ selectively restrains the cytoskeleton and mitochondrial gene expression in osteoclasts. RNA sequencing was performed using the mRNAs extracted from the WT and *Gα*_*13*_^*ΔM/ΔM*^ BMMs stimulated with or without RANKL for five days. (**A**) Gene ontology analysis of RANKL-induced and Gα_13_ deficiency-enhanced genes performed by PANTHER classification system. (**B**) RNAseq–based expression heat map of the RANKL-induced cytoskeleton related genes (Left panel) and mitochondria related genes (Right panel) regulated by Gα_13_ deficiency. The log2 values of the fold changes of TPMs (RANKL condition relative to the non-stimulation control) are shown in the heat map.

To further explore molecular mechanisms by which Gα_13_ acts on osteoclast function, we performed gene expression profiling using high throughput sequencing of RNA (RNAseq) with the WT and *Gα*_*13*_^*ΔM/ΔM*^ osteoclast precursors at baseline and the corresponding osteoclasts after RANKL stimulation for five days to identify genes regulated by Gα_13_. We next performed Gene Ontology (GO) analysis of the up-regulated genes in the RANKL stimulation condition in the *Gα*_*13*_^*ΔM/ΔM*^ osteoclasts. Consistent with the phenotype of the enhanced ability of *Gα*_*13*_^*ΔM/ΔM*^ osteoclasts for adhesion and actin ring organization, GO analysis revealed highly significant upregulation of genes involved in cytoskeleton, ruffle and adhesion (Fig. 6A). We further extracted the gene expression values and found that 307 cytoskeleton-related genes (Fig. 6B, Supplementary Table 1), including *GIT1 and Itgb3*, were significantly elevated by Gα_13_ deficiency. These genome wide analysis data provided another important piece of evidence for the regulatory role of Gα_13_ in cytoskeleton reorganization. The profound effect of Gα_13_ on the cytoskeleton gene expression indicates that Gα_13_ likely functions as a central cytoskeleton regulator. As expected, the genes in charge of osteoclast differentiation, such as *Nfatc1*, *Fos*, *Prdm1*, were not affected by Gα_13_ deficiency (data not shown).

**Figure 7:**
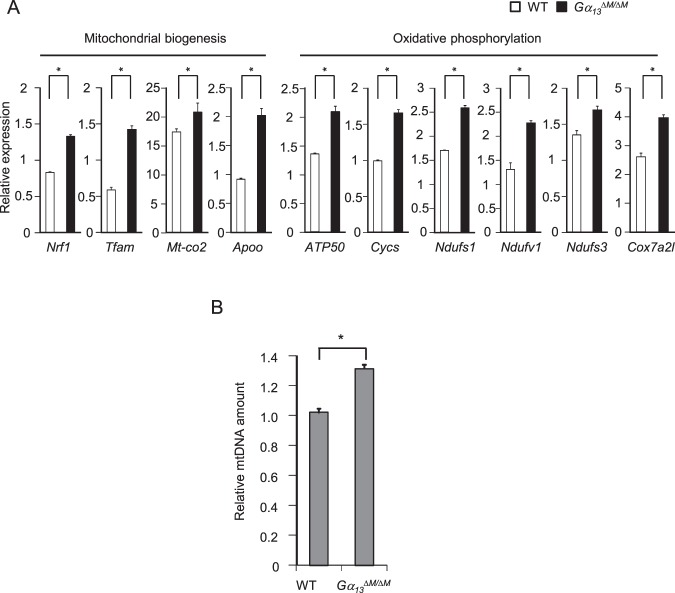
Gα_13_ regulates mitochondrial biogenesis and function and contributes to mitochondrial-mediated actin organization in osteoclasts. (**A**) Quantitative real-time PCR analysis of gene expression responsible for mitochondrial biogenesis and oxidative phosphorylation. (**B**) Relative mitochondrial DNA (mtDNA) amount to chromatin DNA amount. The amount of mitochondrial/chromosol DNA was measured by qPCR analysis for the Cytb gene and Hes1 gene loci using total DNA isolated from the osteoclasts stimulated by RANKL for four days. All data are shown as mean ± S.D. **p < 0.001, n.s., not statistically significant.

Surprisingly, we found that the most enriched gene sets were involved in mitochondrial structural complex and mitochondrial respiratory chain complexes in Gα_13_ deficient osteoclasts (Fig. 6A), indicating a negative regulation of mitochondrial biogenesis and function by Gα_13_. We extracted these genes from the RNA-seq data and confirmed 51 mitochondrial related genes (Figs 6B, 7A) that were highly enhanced in the *Gα*_*13*_^*ΔM/ΔM*^ osteoclasts. These findings obtained from genome wide analysis highlight an exciting and promising novel regulatory network in osteoclasts mediated by Gα_13_ on mitochondrial regulation. Since osteoclast resorption is a highly energy consuming process, the enhanced osteoclast activities in the absence of Gα_13_, including cytoskeleton organization, remodeling and resorption, would require and be supported by increased energy supply from mitochondria. While we were preparing our manuscript, the Zou and Teitelbaum group showed that mitochondria mediated cytoskeleton organization contributes to osteoclast resorptive function^30^. In osteoclasts, we also found that Gα_13_ deficiency enhanced mitochondrial DNA content (Fig. 7B), reflecting an increase in mitochondrial biogenesis. These results, together with the enhanced expression of genes for controlling mitochondrial biogenesis and oxidative phosphorylation in the *Gα*_*13*_^*ΔM/ΔM*^ osteoclasts (Figs 6B, 7A), indicate that Gα_13_ might suppress mitochondrial biogenesis and function, which provide energy supply in osteoclasts. Our findings are corroborated by a recent study demonstrating that Gα_13_ negatively regulates mitochondrial gene expression and function in muscle cells^31^. These results together suggest a novel function for Gα_13_ in the regulation of mitochondria in different cell types. Since mitochondria-provided energy is essential for every cellular function, there is a technical limitation to suppress mitochondrial function while without affecting cellular activity. Thus, we cannot exclude the possibility that the enhanced mitochondrial gene expression is an associated phenomenon along with the enhanced osteoclast function in the Gα_13_ deficient osteoclasts. Nonetheless, although it is unclear whether the regulation is direct or indirectly associated, our results at least indicate that the genes related with mitochondrial biogenesis and function are regulated by Gα_13_.

### Gα_13_ expression is down-regulated in RA and inversely correlated with RA activity

**Figure 8:**
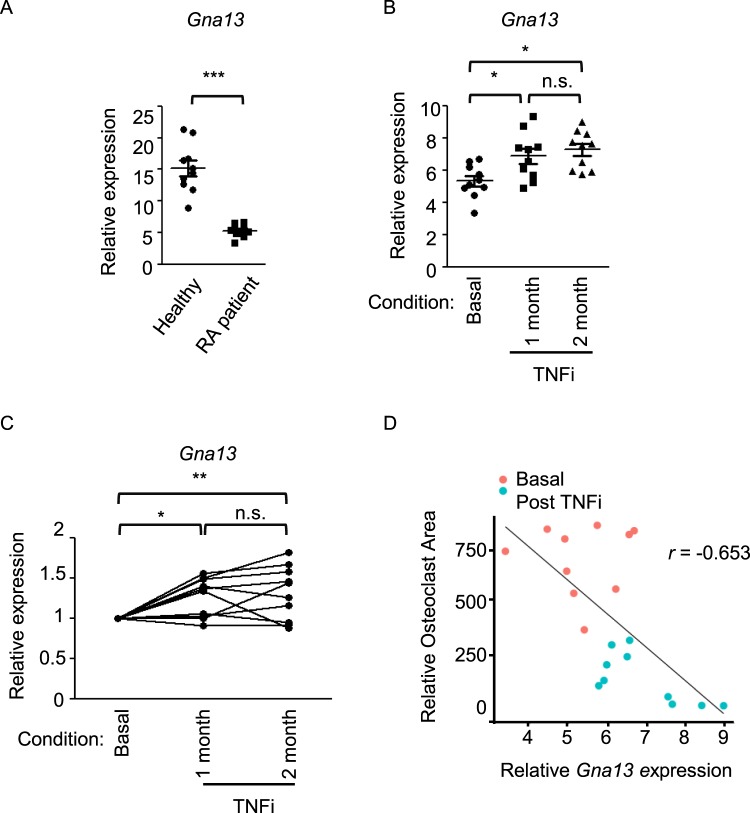
The expression levels of human Gα_13_ are strongly correlated with RA disease. (**A**) Quantitative real-time PCR analysis of human Gα_13_ (Gna13) expression in PBMCs isolated from healthy donors and RA patients. n = 10 for each group. Data are mean ± SEM. (**B**) Quantitative real-time PCR analysis of Gna13 expression in PBMCs isolated from RA patients before (basal level) and after TNFi therapy (Enbrel) for 1 and 2 months. (**C**) Quantitative real-time PCR analysis of the relative expression of Gna13 in PBMCs isolated from the same RA patient prior and after TNFi (Enbrel) for 1 and 2 months. Results were normalized to prior TNFi condition (basal) for each individual patient. (**D**) Scatter plot showing significant inverse correlation between the RA Gna13 expression levels and relative TRAP-positive osteoclast area in the cell cultures of RA CD14-positive PBMC-derived macrophages treated with RANKL (100 ng/ml) for five days. Each dot represents an RA patient in the indicated conditions. Plot was generated by ggplot2 statistical plotting package. Pearson’s r = −0.653. *p < 0.05, **p < 0.01, ***p < 0.001, n.s., not statistically significant.

Having established that Gα_13_ negatively regulates osteoclast resorptive function, we wished to examine whether Gα_13_ expression levels are altered in the RA disease, in which excessive osteoclastic bone resorption is a hallmark of the characteristic pathogenesis of the disease. Similarly as in the mouse system, Gα_13_ expression was highly induced in the human osteoclast precursors derived from CD14(+) peripheral blood monocytes (PBMCs) after a few days of RANKL stimulation (Supplementary Fig. 2). Strikingly, Gα_13_ expression level was decreased in the CD14(+) PBMCs isolated from RA patients compared to healthy donors (Fig. 8A). Our findings suggest that the down-regulated Gα_13_ expression in RA cells can result in the loss of a constraint on osteoclast function based on our data from mouse cells and that this effect could contribute to the pathogenic mechanism mediated by Gα_13_ in the inflammatory bone resorption. Given the importance of TNF in the pathogenesis of RA and the resounding success of TNF blockade therapy (TNFi) in the treatment of RA, we first examined the Gα_13_ effect on the TNF-mediated inflammatory osteoclast formation. In inflammatory conditions, TNF often acts in synergy with RANKL to promote osteoclastogenesis. To mimic the *in vivo* condition, we treated the BMMs with TNF in the presence of RANKL, and found that Gα_13_ deletion also significantly promoted the areas of the TRAP (+) multinucleated osteoclasts (Supplementary Fig. 3), indicating that Gα_13_ plays an inhibitory role in controlling osteoclast spreading in TNF-mediated inflammatory conditions. Furthermore, TNF blockade therapy of RA patients with a humanized antibody that specifically blocks TNF activity (Enbrel) strikingly elevated Gα_13_ expression levels in the PBMCs after one or two month-treatment (Fig. 8B). Moreover, the therapeutic reduction of TNF activity by TNFi significantly increased Gα_13_ expression levels in each individual RA patient after TNFi treatment (Fig. 8C). TNFi therapy decreases TNF activity in the pathogenesis of RA. Therefore, Gα_13_ expression levels were negatively correlated with TNF activity in RA. In parallel, TNFi significantly decreased the osteoclast areas of PBMCs (Fig. 8D). Further statistical analysis revealed a strong inverse correlation (Pearson’s r = −0.653) between the levels of Gα_13_ expression and the sizes of osteoclasts formed in RA PBMC cell cultures (Fig. 8D). Given the feedback effect of Gα_13_ on osteoclast resorptive function, these findings support the idea that low Gα_13_ levels in RA contribute to the pathologic osteoclastic bone erosion. Gα_13_ expression levels are inversely correlated with the RA disease activity and thus could be a predictor of both osteoclast function and disease activity of RA.

## Discussion

The adult skeleton undergoes constant and dynamic remodeling to maintain bone homeostasis, which is tightly regulated by osteoclast-mediated bone resorption and osteoblast/osteocyte-mediated bone formation^17,32,33^. This tightly-regulated balance is disrupted in the chronic inflammation and disease settings such as found in RA and periodontitis, which are some of the most common pathological conditions associated with bone loss. The balance disruption leads to excessive bone resorption and suppressed bone formation^1,3,4^. Clinical inhibition of osteoclast differentiation suppresses osteoclastic resorption, but meanwhile it often reduces osteoclast abundance. This pathological change dampens normal bone remodeling, resulting in insufficient bone formation in multiple disease settings. Recent evidence shows the importance of appropriate maintenance of osteoclast abundance in balancing bone remodeling and stimulating new bone formation^16–19^. Thus, it comes under the spotlight to investigate how to control osteoclast function, which has not been fully understood. In this study, we identify a previously unrecognized role for Gα_13_ in inhibition of osteoclast function. Gα_13_ is selectively highly expressed in multinucleated osteoclasts during osteoclastogenesis. Gα_13_ functions as a brake that restrains the signaling pathways and gene expression to control the ability of osteoclasts in fusion, adhesion, cytoskeletal remodeling and resorption. Deficiency of Gα_13_ leads to an osteoporotic bone phenotype attributed to super spread osteoclasts with enhanced bone resorptive function. Gα_13_ deletion does not affect osteoblast numbers and surfaces in mice (Supplementary Fig. 4). The close correlation between Gα_13_ expression levels, TNF activity and RA disease activity in RA patients suggest that the Gα_13_-pathway represents an attractive therapeutic target to prevent bone destruction in inflammatory diseases associated with excessive bone erosion.

Osteoclast cytoskeleton organization and remodeling, mainly reflected by actin ring and ruffled border formation, is crucial for the osteoclast bone resorptive ability. The defects in the canonical signaling pathways that positively regulate cytoskeleton organization, such as c-Src or Pyk2 deletion, often lead to abnormal actin ring assembly in osteoclasts with contracted morphology and low ability to resorb bone^12–15^. In contrast to these well-studied positive regulatory signals, the feedback regulatory mechanisms involved in cytoskeleton organization and osteoclast function remain poorly understood. The present study identifies Gα_13_ as a newly identified negative regulator of osteoclast cytoskeleton organization and function. Gα_13_ is a RANKL inducible inhibitor that generally reaches high expression levels after RANKL stimulation for a few days, but has marginal expression at early stage of differentiation before three days. The unique expression pattern of Gα_13_ could explain its minimal effects on osteoclast differentiation.

Deletion of Gα_13_ leads to enlarged super spread osteoclasts both *in vitro* and *in vivo*. These Gα_13_ deficient osteoclasts have comparable survivability to WT cells (data not shown), but their abilities of fusion, adhesion, cytoskeleton organization and resorption are significantly enhanced. The underlying mechanisms include the negative regulation of gene expression that is in charge of cell-cell fusion and osteoclast function, c-Src, Pyk2, Erk and FAK mediated signaling and downstream activation of RhoA and ROCK2, as well as a unique mitochondrial regulation by Gα_13_. Thus, the profound regulation of cytoskeleton by Gα_13_ places Gα_13_ in a central knot in the regulatory network mediating osteoclast function. Osteoclasts execute high energy-consuming activities, such as cellular adhesion, migration and bone resorption, all of which require appropriate cytoskeleton organization. To support sufficient energy consumption for these cellular activities, osteoclasts are a mitochondria-rich cell type. Thus, mitochondrial regulation would presumably affect cytoskeleton organization. Indeed, evidence^30^ show that inhibition of mitochondrial function for a short period in osteoclasts almost completely disrupts actin ring structure. In this study, we identified the genome wide regulation of genes involved in mitochondrial biogenesis and function by Gα_13_ in osteoclasts. This Gα_13_ mediated regulation of mitochondria is also observed in muscle cells^31^. Gα_13_ mediated mitochondrial function might contribute to the mechanisms by which Gα_13_ restrains osteoclast cytoskeleton organization and function. It is however a puzzle how exactly Gα13 modulates gene expression in mitochondria. Conversely, cytoskeletal organization can influence mitochondrial network structure and function in certain cells, for example, mitochondria move along microtubules or actin filaments and actin filaments help determine the locations of mitochondria in neurons^34^. Thus, it seems that there is a cross-regulation between mitochondria and cytoskeleton. Although the mechanisms of the bi-directional regulation remain less understood, it is possible that the unique cytoskeletal structure in osteoclasts could regulate mitochondrial distribution and function. These interesting questions have not been explored and future studies would address whether and how mitochondria and cytoskeleton cross talk with each other. Overall, the findings from our genome wide study and another group^31^ indicate a novel function of Gα_13_ that contributes to the regulatory network of mitochondria and open a new avenue to study the biological significance and underlying mechanisms by which Gα_13_ regulates mitochondria and cytoskeleton.

As a G-protein, Gα_13_ can act through small Rho GTPases, such as Rac or RhoA, depending on cell types^20,25^. In osteoclasts, we found that Gα_13_ functions through RhoA to further act on downstream Rock2. The Gα_13_ mediated regulation of the RhoA-Rock2 pathway was also observed in muscle cells^31^. However, it is unclear how Gα_13_ links to different GTPases and regulates their activity differently in various cells, presumably due to the various interactive proteins and guanine-nucleotide exchange factors (GEFs) in different settings^20,25,35^. The upstream GPCRs that connect to Gα_13_, have not been identified in osteoclasts. We searched the GPCRs that were reported to play a part in osteoclasts, such as GPR103, EBI2, GPR68 and GPR55, and found that GPR55 is associated with Gα_13_^36,37^. However, functional analysis *in vitro* could not provide clear evidence that GPR55 and Gα_13_ work in the same pathway in osteoclasts (data not shown). It will be of interest and significance for future studies to investigate and uncover upstream regulators and receptors associated with Gα_13_ in osteoclasts. Our study identified unique downstream factors, pathways and novel mitochondrial mediated mechanisms by which Gα_13_ controls osteoclast cytoskeleton organization and function. Gα_13_, as a novel negative regulator, plays a key role in the maintenance of osteoclast function. Given the deregulated Gα_13_ levels in RA and its correlation with RA disease activity, targeting Gα_13_ and its mediated pathways would provide alternative therapeutic strategies to control osteoclast function and excessive bone resorption associated with many diseases.

## Materials and Methods

### Animal study and analysis of bone phenotype

We generated mice with myeloid specific deletion of Gα_13_ by crossing *Gα13* ^*flox/flox*^ mice^23^ with mice with a lysozyme M promoter-driven Cre transgene on the C57BL/6 background (known as *LysMcre*; The Jackson Laboratory). Gender- and age-matched *Gα*_*13*_^*flox/flox*^ *LysMcre*(+) mice (referred to as *Gα*_*13*_^*ΔM/ΔM*^) and their littermates with *LysMcre*(+) genotype as wild-type controls (referred to as WT) were used for experiments. X-ray analysis was conducted using a high-resolution soft X-ray system (Faxitron Model MX-20) at 26 kV. Micro-computed tomography (µCT) analysis was conducted to evaluate bone volume and 3D bone architecture using a Scanco µCT-35 scanner (SCANCO Medical) as described^38,39^. 2-month-old mouse femora were fixed in 10% buffered formalin and scanned at 6 µm resolution. Proximal femoral trabecular bone parameters were analyzed using Scanco software according to the manufacturer’s instructions and the American Society of Bone and Mineral Research (ASBMR) guidelines. Femur bones were subjected to sectioning, TRAP staining, toluidine blue staining and histological analysis. Mouse serum TRAP was measured with MouseTRAP™ (TRAcP 5b) ELISA (Immunodiagnostic Systems (IDS)) and serum CTX-I was measured using Mouse CTX/Collagen C-Terminal Telopeptide ELISA Kit (LifeSpan BioSciences) according to manufacturer’s instructions. The osteomeasure software (OsteoMetrics, Atlanta, GA, USA) was used for bone histomorphometry using standard procedures according to the program’s instruction. All of the experiments were conducted according to the National Institutes of Health Guide for the Care and Use of Laboratory Animals. All animal studies were approved by the Hospital for Special Surgery Institutional Animal Care and Use Committee (IACUC), and Weill Cornell Medical College IACUC.

### Reagents

Murine M-CSF, murine TNF and soluble human RANKL were purchased from PeproTech (Rochy Hill, NJ, USA). Recombinant Fibronectin was purchased from Sigma-Aldrich. Alexa Fluor^®^ 488 phalloidin (A12379) was purchased from Invitrogen (Carlsbad, CA, USA).

### Cell culture

To obtain bone marrow macrophages (BMMs), mouse bone marrow cells were harvested from tibiae and femora of age and gender-matched mutant and control mice and cultured for 3 days in α-MEM medium (Thermo Fisher Scientific) with 10% FBS (Atlanta Biologicals), glutamine (2.4 mM, Thermo Fisher Scientific), Penicillin-Streptomycin (Thermo Fisher Scientific) and CMG14-12 supernatant (condition medium, CM), which contained the equivalent of 20 ng/ml of rM-CSF and was used as a source of M-CSF as described^38^. The attached BMMs were scraped, seeded at a density of 4.5 × 10^4^/cm^2^, and cultured in α-MEM medium with 10% FBS, 1% glutamine and CM for overnight. Except where stated, the cells were then treated with or without optimized concentrations of RANKL (40 ng/ml) or TNF (40 ng/ml) in the presence of CM for times indicated in the figure legends. Culture media were exchanged every three days. Human osteoclast cultures were performed as described previously^40^. Briefly, peripheral blood mononuclear cells (PBMCs) from whole blood of healthy volunteers or RA patients were isolated by density gradient centrifugation using Ficoll (Invitrogen Life Technologies, Carlsbad, CA). CD14+ cells were purified from fresh PBMCs using anti-CD14 magnetic beads (Miltenyi Biotec, Auburn, CA) as recommended by the manufacturer. Human monocytes were cultured in α-MEM medium with 10% FBS in the presence of M-CSF (20 ng/ml; PeproTech, Rocky Hill, NJ) for 2 days to obtain monocyte-derived macrophages, which were further cultured with RANKL for osteoclast differentiation. The RA PBMCs were from RA patients (age ≥ 18 and <70 years) who fulfilled American College of Rheumatology (ACR) 2010 RA classification criteria with disease duration <5 years and were under TNFi therapy for the first time (Enbrel, 25 mg weekly). Experiments with human cells were approved by Nanfang Hospital (China) and the Hospital for Special Surgery (USA) Institutional Review Board. Informed consent (PBMC collection) was obtained from all healthy volunteers and RA patients. All experiments were performed in accordance with relevant guidelines and regulations. TRAP staining was performed with an acid phosphatase leukocyte diagnostic kit (Sigma-Aldrich) in accordance with the manufacturer’s instructions.

### Actin cytoskeleton reorganization assay

2 × 10^4^ WT or *Gα*_*13*_^*ΔM/ΔM*^ BMMs were cultured on 96 well plates for three days or 6 × 10^3^ WT or *Gα*_*13*_^*ΔM/ΔM*^ BMMs on dentin slices (0.4 cm in diameter) for six days with CM (equivalent of 20 ng/ml of rM-CSF) and 40 ng/ml RANKL. The cells were then starved in the culture medium without FBS, M-CSF and RANKL for 5 hours, and were re-stimulated with 20 ng/ml M-CSF and 40 ng/ml RANKL for 0, 5, 15 or 30 minutes as indicated in figure legends. Phalloidin staining was performed in accordance with manufacturer’s instructions. Briefly, the cells were fixed with 4% PFA/PBS for 10 min at room temperature (RT) and washed with PBS. Then, the cells were permeabilized in 0.5% Triton X-100/PBS for 5 min followed by blocking with 1% BSA/PBS for 20 min at RT. The cells were then incubated with Alexa Fluor^®^ 488 phalloidin (Invitrogen, 1:500 dilution) for 20 minutes. After washing with PBS, fluorescent signals were detected with a fluorescent microscope. The osteomeasure software (OsteoMetrics, Atlanta, GA, USA) was used for the quantitative analysis of actin ring/podosome belt formation in the cell cultures using standard procedures according to the program’s instruction.

### *In vitro* adhesion assay

To prepare fibronectin-coated plates, 6 well plates were incubated with 1 ml of fibronectin (10 μg/ml) per each well for 2 hours at 37 °C. The plates were then washed with PBS for 3 times and were ready for adhesion assay. 8 × 10^6^ WT or *Gα13*^*ΔM/ΔM*^ BMMs were cultured with CM (equivalent of 20 ng/ml of rM-CSF) and 40 ng/ml RANKL on 10 cm culture dishes for 3 days. The cells were then starved in the culture medium without FBS, M-CSF and RANKL for 5 hours, and were lifted by scraper. 2 × 10^6^ cells were then plated on the fibronectin-coated 6 well tissue culture plates (attached cells) or left in αMEM as suspension conditions for 15 min or 30 min, followed by immunoblot analysis or GTP-RhoA pull down assay.

### Mineral resorption pit assay

The mineral resorption activity of osteoclasts was examined using 96-well Corning Osteo Assay Surface Plates (Sigma-Aldrich). BMMs were plated at a seeding density of 2 × 10^4^ per well and incubated with CM (equivalent of 20 ng/ml of rM-CSF) and RANKL (40 ng/ml) for 8 days, with medium exchange every day once mature round shaped-osteoclasts were observed. After removing cells with 10% bleach solution, the minerals were stained with von kossa to visualize the formation of resorptive pits. The resorptive area was analyzed using ImageJ (National Institutes of Health, Bethesda, MD, USA).

### Reverse transcription and real-time PCR

For quantification of mRNA, reverse transcription and real-time PCR were performed as previously described^40^. Briefly, DNA-free RNA was obtained using the RNeasy Mini Kit (QIAGEN) with DNase treatment, and total RNA was reverse transcribed using a First Strand cDNA Synthesis Kit (Thermo Fisher Scientific, Waltham, MA, USA). Real-time PCR was performed in triplicate using Fast SYBR^®^ Green Master Mix and the QuantStudio5 Real-Time PCR System (Applied Biosystems, Foster City, CA) following the manufacturer’s protocol. Gene expression was normalized relative to the gene expression levels of Glyceraldehyde-3-phosphate dehydrogenease (*Gapdh*). The primer sequences are available upon request.

### RNA-seq and Bioinformatics analysis

Total RNA was extracted using RNeasy Mini Kit (QIAGEN) following the manufacturer’s instructions. True-seq RNA Library preparation kits (Illumina) were used to purify poly-A+ transcripts and generate libraries with multiplexed barcode adaptors following the manufacturer’s instructions. All samples passed quality control analysis using a Bioanalyzer 2100 (Agilent). RNA-seq libraries were constructed per the Illumina TrueSeq RNA sample preparation kit. High-throughput sequencing was performed using the Illumina HiSeq 4000 in the Weill Cornell Medical College Genomics Resources Core Facility. RNA-seq reads were aligned to the mouse genome (mm10) using STAR^41^. HTseq^42^. was subsequently used to count reads in features and then EdgeR^43^ was used to estimate the transcript abundances as TPM (transcripts per million) values. Heatmaps were generated by the pheatmap package in R. Gene Ontology (GO) analysis was performed with Panther Classification System^44,45^ input with the RANKL-regulated genes that were more highly expressed in *Gα13*^*ΔM/ΔM*^ cells than WT cells (≥1.5 fold). Gα13 regulated pathways by the GO analysis were ranked based on the *p* values. *p* values were calculated following the program’s instructions. RNA-seq data (accession #GSE116550) have been deposited in NCBI’s Gene Expression Omnibus (http://www.ncbi.nlm.nih.gov/geo/query/acc.cgi?acc=GSE116550).

### Immunoblot analysis

Total cell extracts were obtained using lysis buffer containing 150mMTris-HCl (pH 6.8), 6% SDS, 30% glycerol, and 0.03% Bromophenol Blue; 10% 2-ME was added immediately before harvesting cells. Cell lysates were fractionated on 7.5% SDS-PAGE, transferred to Immobilon-P membranes (Millipore), and incubated with specific antibodies. Western Lightning plus-ECL (PerkinElmer) was used for detection. For immunoblotting, anti-NFATc1 (556602, 1:1000 dilution) was from BD Biosciences. Anti-Blimp1 (sc-47732, 1:1000 dilution), anti-Gα13 (sc-410, 1:500 dilution), anti-RhoA (sc-418), and anti-p38α (sc-535, 1:1000 dilution) were purchased from Santa Cruz Biotechnology (Dallas, TX, USA). Anti-pyk2 (06-559, 1:1000 dilution) was from EMD Millipore. Anti-phospho-Pyk2 (Try402) (#3291, 1:1000 dilution), anti-phospho-Akt (Ser473) (#9271, 1:1000 dilution), anti-phospho-Akt (Thr408) (#2965, 1:1000 dilution), anti-Akt (#9272, 1:1000 dilution), anti-phospho-ERK (Ser473) (#9101, 1:1000 dilution), anti-ERK (#9102, 1:1000 dilution), anti-phospho-Src family (Tyr416) (#2101, 1:1000 dilution) were purchased from Cell Signaling Technology Inc. (Danvers, MA, USA). Anti- phospho-Rock2 (Ser1366, 1:500 dilution) is from GeneTex.

### Purification of GST-PBD protein

The GST-PBD plasmid^27^ was purified as described^46^. Briefly, fusion protein containing RhoA-binding domain Rhotekin (RhBD, amino acids 7–89) fused with GST were produced in *Eschericia coli* BL21 cells. After isopropylthigalactoside (IPTG) induction for 4 hrs at 30 °C, pellets of bacteria were resuspended in Bacterial lysis buffer containing 50 mM Tris–HCl, pH 7.5, 150 mM NaCl, 5 mM MgCl_2_, 1 mM EDTA, 1 mM dithiothreitol (DTT), 1 mM phenylmethylsulfonyl fluoride (PMSF), and 1 μg/mL aprotinin and then sonicated. Cell lysates were incubated with GST beads (ThermoFisher) for 2 hrs at 4 °C. The beads were wash 5 times with washing buffer containing 50 mM Tris–HCl, pH 7.5, 150 mM NaCl, 5 mM MgCl_2_, 1 mM EDTA, 1 mM dithiothreitol (DTT), 1 mM PMSF, and 1 μg/mL aprotinin. The beads were aliquoted in wash buffer with 10% v/v glycerol (1:1 slurry) and stored at −80 °C.

### Affinity- precipitation of cellular GTP-RhoA (pulldown assay)

The suspension and attached osteoclasts from adhesion assay were lysed in a buffer containing 50 mM Tris/HCl, pH 7.5, 1 mM EDTA, 500 mM NaCl, 10 mM MgCl_2_, 1% Triton X-100, and protease inhibitors (4 µg/ml leupeptin, 5 µg/ml apropeptin, 1 mM PMSF, 5 mM NaF, 5 mM sodium orthovanadate and protease inhibitor cocktail). Cell lysates were clarified by centrifugation at 15 000 g at 4 °C for 10 min. Equal volumes of lysates were incubated with 50 µg GST–RBD fusion protein at 4 °C for 1 hr while rotating. After pulldown, the beads were centrifuged at 250 g for 2 min and 4 °C and then washed five times with a buffer containing 50 mM Tris/HCl, pH 7.5, 150 mM NaCl, 10 mM MgCl_2_, 1% Triton X-100, 10 µg/ml leupeptin, 10 µg/ml apropeptin and 1 mM PMSF. The pulled down active RhoA proteins were analyzed by SDS-PAGE followed by immunoblotting against RhoA (Santa Cruz Biotechnology, sc-418). ImageJ was used to quantify the immunoblot band density that reflects the amount of proteins, and the relative amount of GTP-bound RhoA was normalized to the total amount of RhoA in cell lysates for the comparison of RhoA activity in different samples.

### Statistical analysis

Statistical analysis was performed using Graphpad Prism® software. The two-tailed Student’s *t* test was applied when there were only two groups of samples. In the case of more than two groups of samples, one-way ANOVA was used with one condition, and two-way ANOVA was used with more than two conditions. ANOVA analysis was followed by Post hoc Bonferroni’s correction for multiple comparisons. *p* < 0.05 was taken as statistically significant; *p value < 0.05, **p value < 0.01 and ***p value < 0.001. Data are presented as the mean ± SD as indicated in the figure legends.

## Supplementary information

Supplemental figure
