# Supplementary material for: G-protein Gα13 functions as a cytoskeletal and mitochondrial regulator to restrain osteoclast function
Source: Sci Rep. 2019 Mar 12;9:4236. doi: 10.1038/s41598-019-40974-z (PMC6414604; doi:10.1038/s41598-019-40974-z)
Supplement: Supplementary file 1 — Supplemental figure [file 41598_2019_40974_MOESM1_ESM.pdf]

SUPPLEMENTARY INFORMATION

G-protein Gα_{13} functions as a cytoskeletal and mitochondrial regulator to restrain osteoclast function

Shinichi Nakano, Kazuki Inoue, Cheng Xu, Zhonghao Deng,
Viktoriya Syrovatkina, Gregory Vitone, Liang Zhao, Xin-Yun Huang,
Baohong Zhao

Supplementary Figure 1

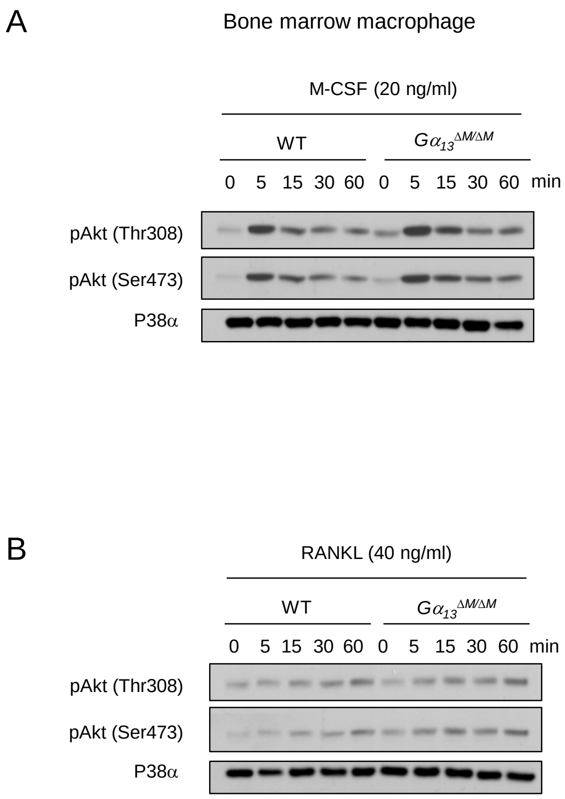

Supplementary Figure 1. Immunoblot analysis of the expression levels of phospho-Akt (Thr308 or Ser473) in the WT and $G\alpha_{13}^{\Delta M/\Delta M}$ BMMs treated with M-CSF (20 ng/ml) for the indicated times (A) or the WT and $G\alpha_{13}^{\Delta M/\Delta M}$ osteoclasts treated with RANKL (40 ng/ml) at the indicated times. p38α was used as a loading control.

Supplementary Figure 2

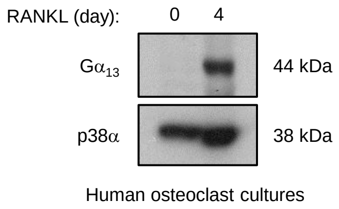

Supplementary Figure 2. Immunoblot analysis of $G\alpha_{13}$ expression induced by RANKL in human CD14-positive PBMC-derived macrophage cultures.

Supplementary Figure 3

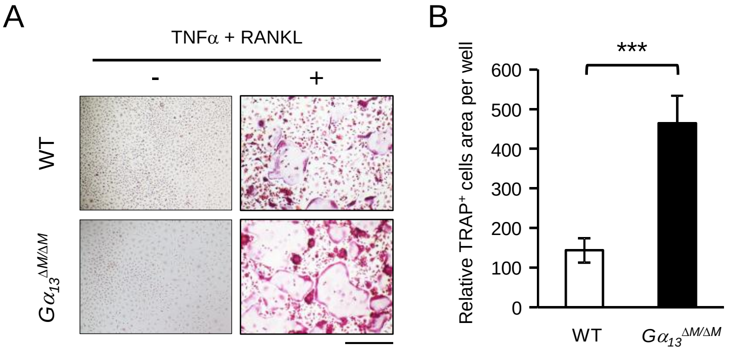

Supplementary Figure 3. Gα_{13} deficiency enhances the size of osteoclasts primed by TNF. (A) TRAP staining and (B) quantification of the relative TRAP positive MNC areas in the WT and $G\alpha_{13}^{\Delta M/\Delta M}$ BMMs primed by TNF and followed by co-stimulation of TNF and RANKL for four days. ***p<0.001.

Supplementary Figure 4

A

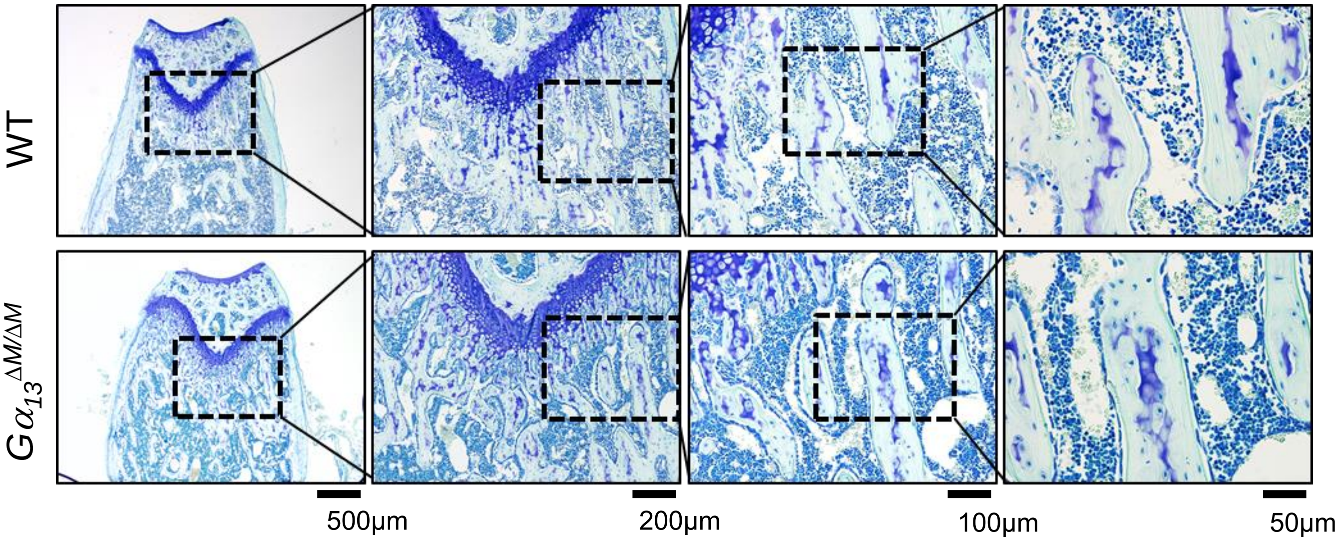

B

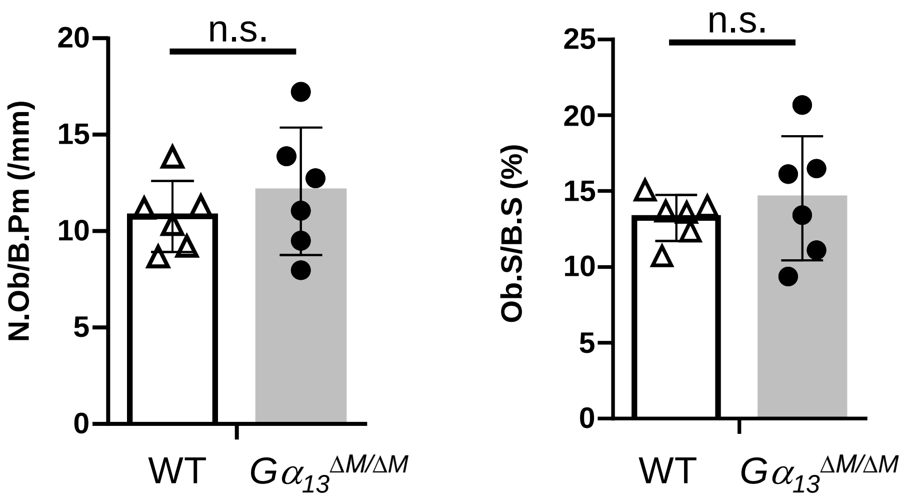

Supplementary Figure 4. Gα_{13} deficiency does not affect osteoblast numbers and surfaces in mice. (A) Toluidine blue staining of femurs. Osteoblasts are stained blue on the bone surface, and (B) quantification of osteoblast numbers and surfaces relative to bone surface. N.Ob/B.Pm: Number of osteoblasts per bone perimeter. Ob.S/B.S: Osteoblast surface per bone surface. N=6 in each group. n.s., not statistically significant.

Supplementary Figure 5

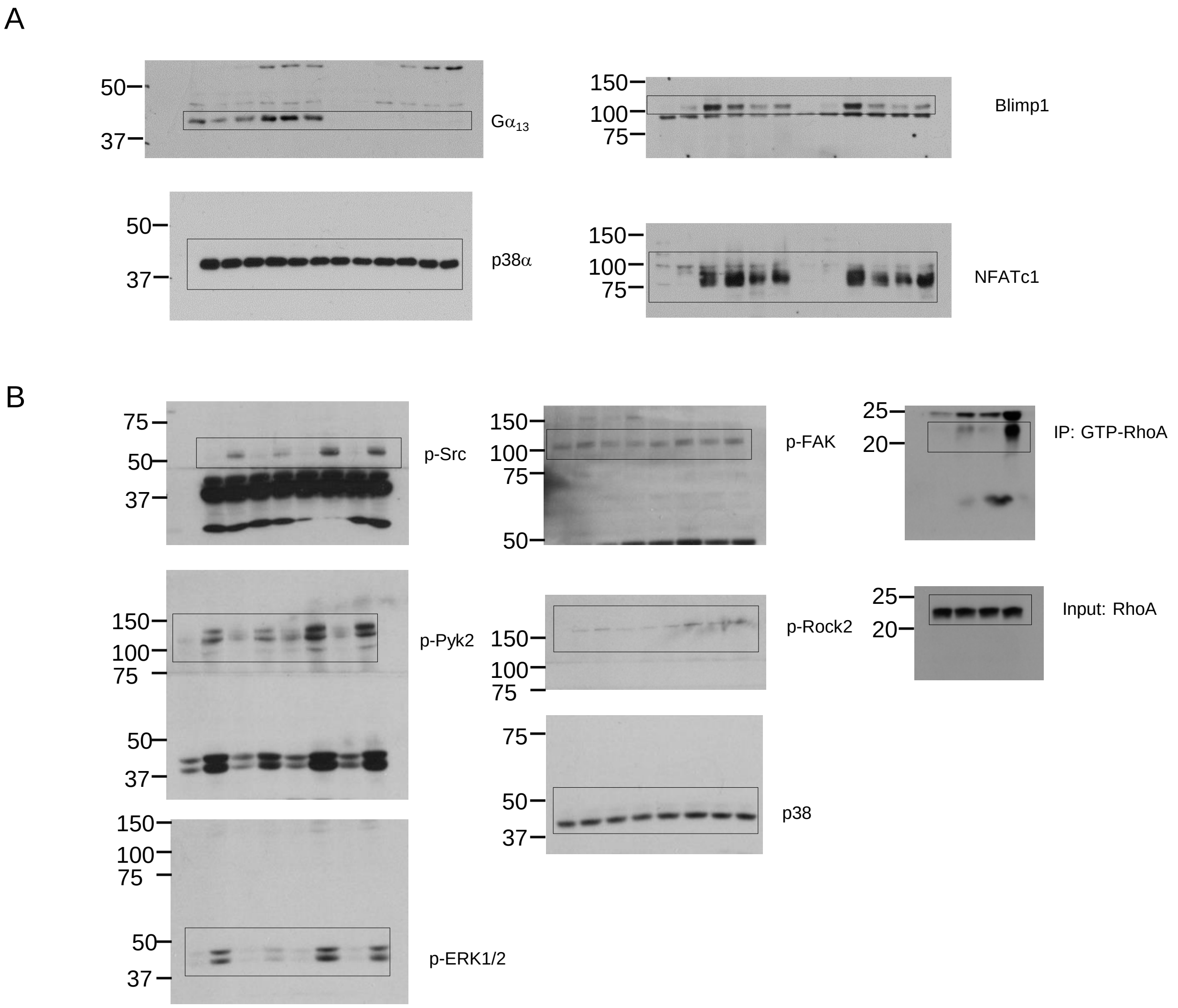

Supplementary Figure 5. (A) Uncropped western blots related to Fig1A and Fig4B. (B) Uncropped western blots related to Fig5A and B.

1	Arhgef18	41	Cep55	81	Myo1e	121	Trmt10a	161	Stk38l	201	Actr3	241	Dynll1	281	Tctex1d2
2	Mns1	42	Tubb6	82	Ccdc181	122	Nicn1	162	Kbtbd8	202	Myo10	242	Rap1gap2	282	Cluap1
3	Rpl10a	43	Cadm1	83	Cit	123	Trim59	163	Sclt1	203	Ndc1	243	Shcbp1	283	Cdca3
4	Mid1	44	Klhl12	84	Tuba1c	124	Wdr34	164	Flnb	204	Syne1	244	Pxk	284	Nme2
5	Camsap3	45	S100a9	85	Fam161a	125	Ccdc28b	165	Sgol1	205	Gsn	245	Cenpj	285	Arl8b
6	Wdr35	46	Rilpl1	86	Mkks	126	Tmem201	166	Cenpf	206	Vcl	246	Aim1	286	Msn
7	Tpt1	47	Ska1	87	Tnfrsf12a	127	Dync2h1	167	Tti2	207	Katnb1	247	Cc2d2a	287	Mad1l1
8	Cetn4	48	Rmdn2	88	Xrcc2	128	Trim36	168	Rassf1	208	Nsun2	248	Ttbk2	288	Cetn2
9	Tctex1d4	49	Ska2	89	Vim	129	Ajuba	169	Sra1	209	Mesdc1	249	Itga5	289	Cep44
10	Arap3	50	Rab3ip	90	Map1lc3a	130	Ezr	170	Spata7	210	Sntb2	250	Arhgap6	290	Lrp1
11	Myo19	51	Capn5	91	Hyls1	131	Top2a	171	Polr3h	211	Ttll4	251	Atf4	291	Tpgs1
12	Gja1	52	Tacc2	92	Kif22	132	Clic4	172	Gtl3	212	Rps16	252	Rapgef6	292	Lama3
13	Cav1	53	Stil	93	Abcb4	133	Narf	173	Dync1li2	213	Gapdh	253	Prpf19	293	Klhl2
14	Mical3	54	Igf2bp2	94	Mapk8	134	Terf1	174	Atp6v1b2	214	Cenpe	254	Ssh1	294	Arf1
15	Krt10	55	Ccnb1	95	Mid1ip1	135	Cep57l1	175	Itgb3	215	Birc5	255	Ccdc6	295	Mecp2
16	Cd9	56	Ttc28	96	Git1	136	Fam110b	176	Anln	216	Ranbp1	256	Fam110a	296	Espl1
17	Cttn	57	Tubb2b	97	Stau2	137	Fbxo5	177	Lzts2	217	Nlrc3	257	Usp2	297	Fkbp4
18	Slc16a1	58	Tubb4a	98	Mpp7	138	Brca1	178	Nckap1	218	Haus7	258	Sh3kbp1	298	Tlk2
19	Scnn1a	59	Pip5k1b	99	Rps10	139	Gpsm2	179	Kif20a	219	Racgap1	259	Leo1	299	Rab39
20	Ubxn11	60	Pcgf5	100	Pstpip1	140	Ttc30a1	180	Ofd1	220	S100a6	260	Kitl	300	Nme1
21	Pvrl1	61	Mmp14	101	Palld	141	Capg	181	Dapk3	221	Ric8b	261	Camk2d	301	Ccnb2
22	Arhgap26	62	Tmub1	102	Tes	142	Cdc42bpa	182	Tmem63b	222	Cdk5rap2	262	Cep290	302	Abi1
23	Cenpq	63	Ccne1	103	Mical1	143	Kif23	183	Ttl	223	Actn1	263	Ska3	303	Lims1
24	Amot	64	Ckap4	104	Tpm1	144	Atp6v1d	184	Rassf3	224	Ccdc88a	264	Gramd3	304	Rmdn1
25	Smtn	65	Itgav	105	Ndrg1	145	Fblim1	185	Cd97	225	Map2k6	265	Ttc8	305	Lancl2
26	Cdc42ep2	66	Tubd1	106	Snx10	146	Sipa1l3	186	Rplp2	226	Alkbh2	266	Eef1a1	306	Aurka
27	Trip6	67	Pde4dip	107	Aldoa	147	Gbp2	187	Pard6a	227	Dctn3	267	Crocc	307	Lrif1
28	Fgr	68	Myo1b	108	Rsph3a	148	Ift81	188	Actn4	228	Apbb1ip	268	Ift74		
29	Clmp	69	Rpl12	109	Kif24	149	Myc	189	Aaas	229	Rac2	269	P4hb		
30	Capn10	70	Bloc1s2	110	Plk1	150	Rps7	190	Rif1	230	Bsg	270	Tubg1		
31	Pdlim7	71	Nckap5l	111	Nusap1	151	Lmna	191	Cd59b	231	Cdc16	271	Bora		
32	Strbp	72	Coro2a	112	Kif14	152	Arhgap35	192	Nedd1	232	Kif20b	272	Ywhaz		
33	Ak1	73	Id1	113	Anxa1	153	Pls3	193	S100a8	233	Ywhae	273	Apc		
34	Smad7	74	Rnd3	114	Mzt1	154	Tiam1	194	Dpysl2	234	Mfn2	274	Shmt2		
35	Sh3yl1	75	Ccsap	115	Mapk6	155	Dlgap5	195	Hspa9	235	Trip10	275	Cep170b		
36	Cdc7	76	Pbxip1	116	Mllt4	156	Nf2	196	Dtl	236	Pgm2	276	Dsn1		
37	Cdc20	77	Tuba4a	117	Hspb11	157	Acaca	197	Pabpc1	237	Ect2	277	Rragd		
38	Myo1d	78	Uaca	118	Hsp90aa1	158	Tpx2	198	Slc25a5	238	Rhof	278	Kif1c		
39	Rai14	79	Hook1	119	Fnbp1l	159	H2afx	199	Rras2	239	G3bp1	279	Cdk2ap2		
40	Slc4a2	80	Iqcb1	120	Flrt2	160	Anxa2	200	Ppp2r3c	240	Prc1	280	Kifc3		

Supplementary Table 1. List of upregulated cytoskeleton related genes in $G\alpha_{13}^{\Delta M/\Delta M}$ osteoclasts.
